# The Oligo–Miocene closure of the Tethys Ocean and evolution of the proto-Mediterranean Sea

**DOI:** 10.1038/s41598-020-70652-4

**Published:** 2020-08-14

**Authors:** Adi Torfstein, Josh Steinberg

**Affiliations:** 1 The Fredy and Nadine Herrmann Institute of Earth Sciences, The Hebrew University of Jerusalem, 91904 Jerusalem, Israel; 2 The Interuniversity Institute of Marine Sciences of Eilat, Eilat, Israel; 3 Ratio Oil Exploration, Tel Aviv, Israel

**Keywords:** Tectonics, Palaeoceanography, Palaeoclimate

## Abstract

The tectonically driven Cenozoic closure of the Tethys Ocean invoked a significant reorganization of oceanic circulation and climate patterns on a global scale. This process culminated between the Mid Oligocene and Late Miocene, although its exact timing has remained so far elusive, as does the subsequent evolution of the proto-Mediterranean, primarily due to a lack of reliable, continuous deep-sea records. Here, we present for the first time the framework of the Oligo–Miocene evolution of the deep Levant Basin, based on the chrono-, chemo- and bio- stratigraphy of two deep boreholes from the Eastern Mediterranean. The results reveal a major pulse in terrigeneous mass accumulation rates (MARs) during 24–21 Ma, reflecting the erosional products of the Red Sea rifting and subsequent uplift that drove the collision between the Arabian and Eurasian plates and the effective closure of the Indian Ocean-Mediterranean Seaway. Subsequently, the proto-Mediterranean experienced an increase in primary productivity that peaked during the Mid-Miocene Climate Optimum. A region-wide hiatus across the Serravallian (13.8–11.6 Ma) and a crash in carbonate MARs during the lower Tortonian reflect a dissolution episode that potentially marks the earliest onset of the global middle to late Miocene carbonate crash.

## Introduction

Global climate oscillations during the Miocene reflect major events in the Earth’s history such as the closure of the Indian Ocean-Mediterranean Seaway (IOMS), Mid-Miocene Climate Optimum (MMCO), and the glaciation of Antarctica^1^. While these events are widely reported in marine records globally, their dynamics are not well known in mid-latitudinal settings, such as the proto-Mediterranean (PM) Sea, which formed following the closure of the Tethys Ocean. Throughout the Oligocene and Miocene, significant changes in the paleogeography of the northern Arabian Peninsula were driven by the Eurasia-Arabia collision along the Bitlis–Zagros thrust zone and the subsequent uplift and sub-aerial exposure of an extensive area^2–4^, resulting in the disconnection of the Mediterranean basin from the Mesopotamian basin (Indian Ocean).

**Figure 1:**
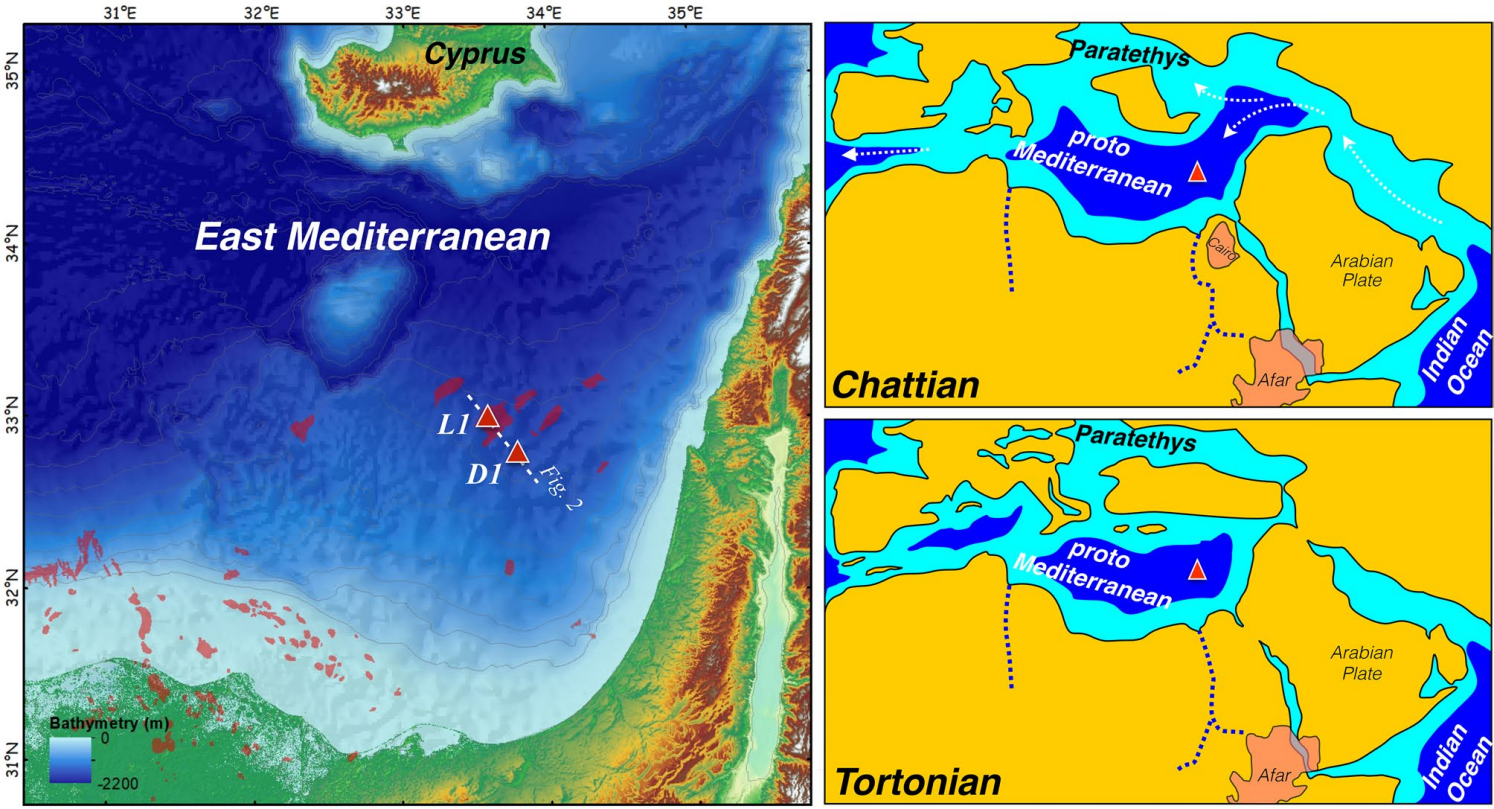
Location map of boreholes in present day (left panel; ArcMap software v10.5, https://www.esri.com/en-us/arcgis/products/arcgis-pro/overview) and during the Chattian and Tortonian (upper and lower right panels, respectively). The site of the Leviathan-1 (L1) and Dolphin-1 (D1) drill holes is marked by a red triangle. Miocene maps reconstructed from a compilation of previous studies^4,19,27,33,48,49^. Red polygons in the left panel mark the location of gas fields, red-shaded areas in right panels mark the Cairo and Afar Plumes, and white arrows mark surface water circulation patterns.

**Figure 2:**
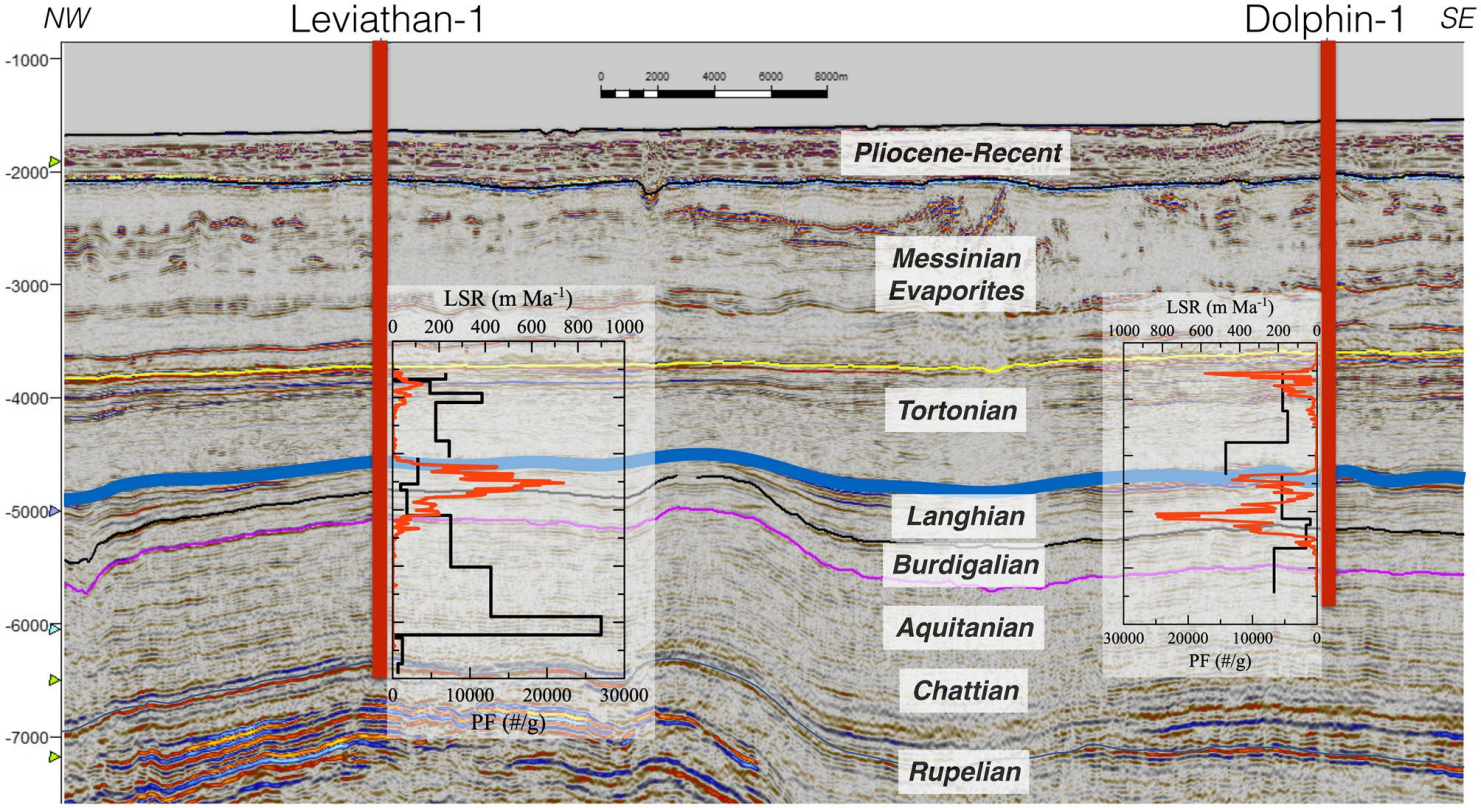
Seismic reflection and well section crossing through the study area. Location of line can be seen in Fig. 1. The contacts between the geologic units are marked as colored horizons. In addition, the Linear Sedimentation Rates (LSR) and Planktonic Foraminifera (PF, red curves) shell contents are plotted along the vertical section of each well.

The Levant Basin, located between the Levant continental margin and the Eratosthenes Seamount, evolved during the Permian–Triassic as part of the regional opening of the neo-Tethys^5,6^. Current reconstructions of the evolution of this basin are derived from regional syntheses of seismic data, together with studies of outcrops and/or subsurface sediment sequences in continental environments^4,7–9^. Additional estimates of the tectonic evolution of the region are derived from studies of provenance and formation ages of detrital minerals^10,11^. Yet, only very few marine sedimentary records cover the Oligo–Miocene period in this region in its entirety, and available knowledge is typically at very coarse temporal resolution. The Leviathan-1 and Dolphin-1 wells (Figs. 1, 2) have uniquely penetrated a thick sedimentary sequence representing the full span of the Oligocene and Miocene, and their litho-, chrono- and chemo- stratigraphy are presented here for the first time, shedding light on the history of this region. The newly discovered, exceptionally thick, late Tertiary sequence of the Levant Basin^3,12^ provides the opportunity to close critical gaps of knowledge in our understanding of the history of the closure of the Tethys Ocean and the depositional environment in the Levant Basin during the Miocene, together with the reconstruction of the marine environment in this region, and understanding its relation to globally occurring changes in climate and ocean circulation. Added interest in understanding the history of this region stems from recent findings of vast hydrocarbon reservoirs in the Levant Basin^13^.

Here, we present a new biostratigraphy-based age model with significantly more detail than the previously available age models in this region^3^ and combine it with the measured carbonate contents, elemental concentrations and planktic and bentic foraminifera abundances, to reconstruct the history of the PM and discuss the findings in the context of tectonic and global climatic processes. Sample cuttings, which are frequently dismissed due to suspected drill mud contamination, are shown here to faithfully record the primary lithological and chemical properties of the well sequences (Fig. S1), and thus, open up the possibility for large scale investigations of multiple wells whose sample cuttings are available but have thus far not been investigated and incorporated in a regional model.

## Results and discussion

### Basin sediment fill

**Figure 3:**
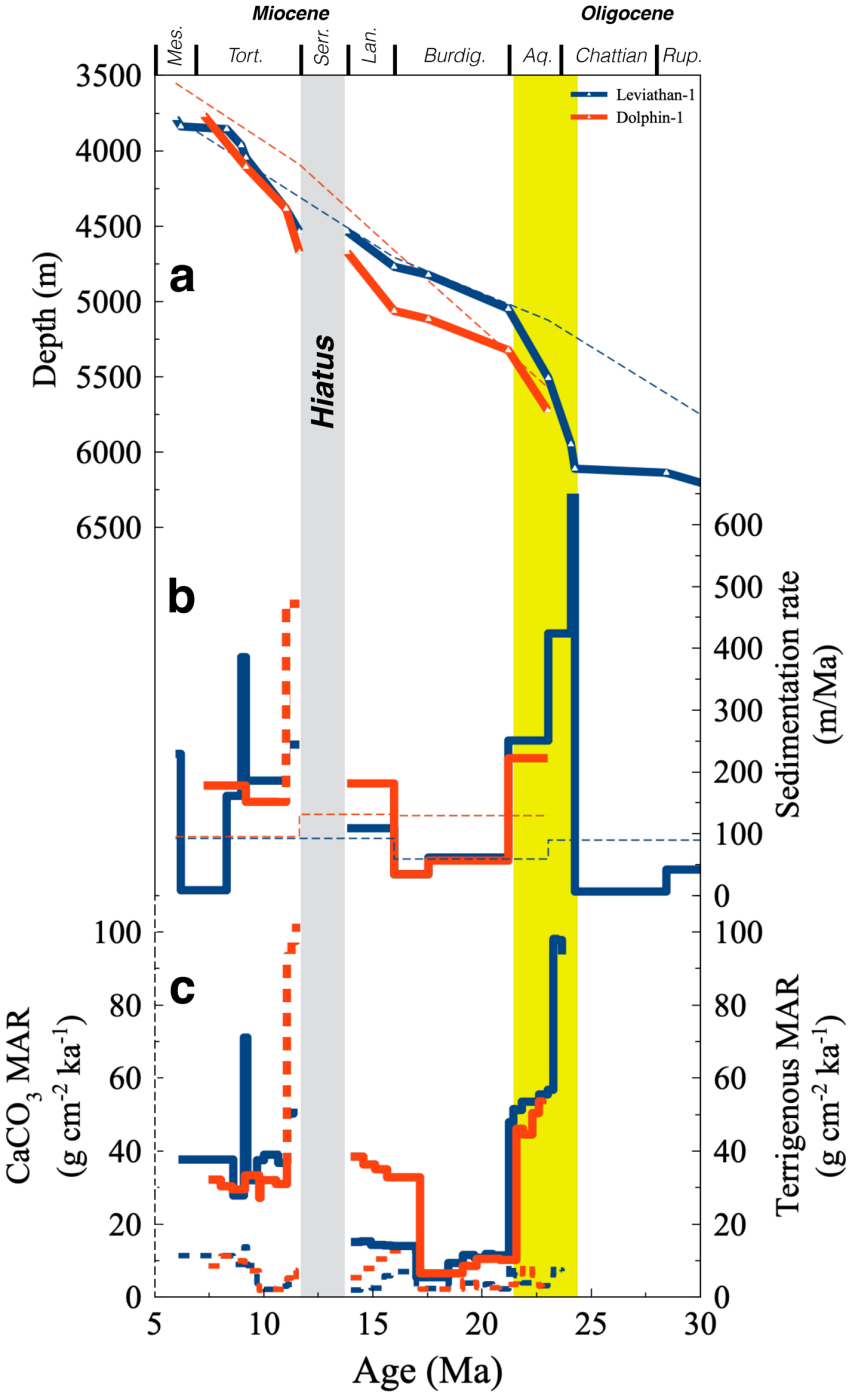
Age models and sedimentation rates in the Leviathan-1 and Dolphin-1 drill holes. (**a**) Published (dashed curves)^3^ and new age models (this work), (**b**) linear sedimentation rates based on published (dashed curves)^3^ and new age models (this work), (**c**) terrigenous and CaCO_3_ (continuous and dashed curves, respectively) Mass Accumulation Rates (MARs). Note the order of magnitude increase in sedimentation between ~ 24 and 21 Ma, interpreted to reflect the IOMS closure in response to the coeval collision of the Arabian and Eurasian plates, rifting and volcanism along the Red Sea. This pulse is primarily driven by an increase in terrigenous fluxes. A sharp albeit brief increase in LSRs and MARs immediately above the Serravalian hiatus in the Dolphin-1 well (marked by dashed curve), is probably a bias of boundary conditions and choice of the exact timing the hiatus ends, and is therefore not discussed.

The two wells, located ~ 30 km from each other (Figs. 1, 2), display cyclic shifts between sand-dominated intervals, marl, clay, and a thick halite interval associated with the Messinian Salinity Crisis^14,15^. Previously, it has been argued that the large amounts of terrigenous material that flowed basinward since the exposure of the Arabian platform coeval with the uplift of East Africa, increased sedimentation rates in the Levant Basin from ~ 5 m/Myr in the Paleocene to ~ 100 m/Myr since the Late Eocene^3^ (Fig. 3). The new chronology and calculated MARs reveal a continuous record of deposition between the Rupelian until the Messinian, with the exception of a prominent hiatus during the Serravallian. It further provides significantly more detail than has been available and implies that while MARs did indeed increase beginning in the early Oligocene, the most significant change occurred during the late Chattian and Aquitanian (~ 24–21 Ma), when values peaked at 50–100 g cm^−2^ ka^−1^, two orders of magnitude higher than pre Oligocene MARs^16^ (Fig. 3 and *Methods*). Middle and late Miocene MARs were mostly in the range of ~ 10–50 g cm^−2^ ka^−1^.

**Figure 4:**
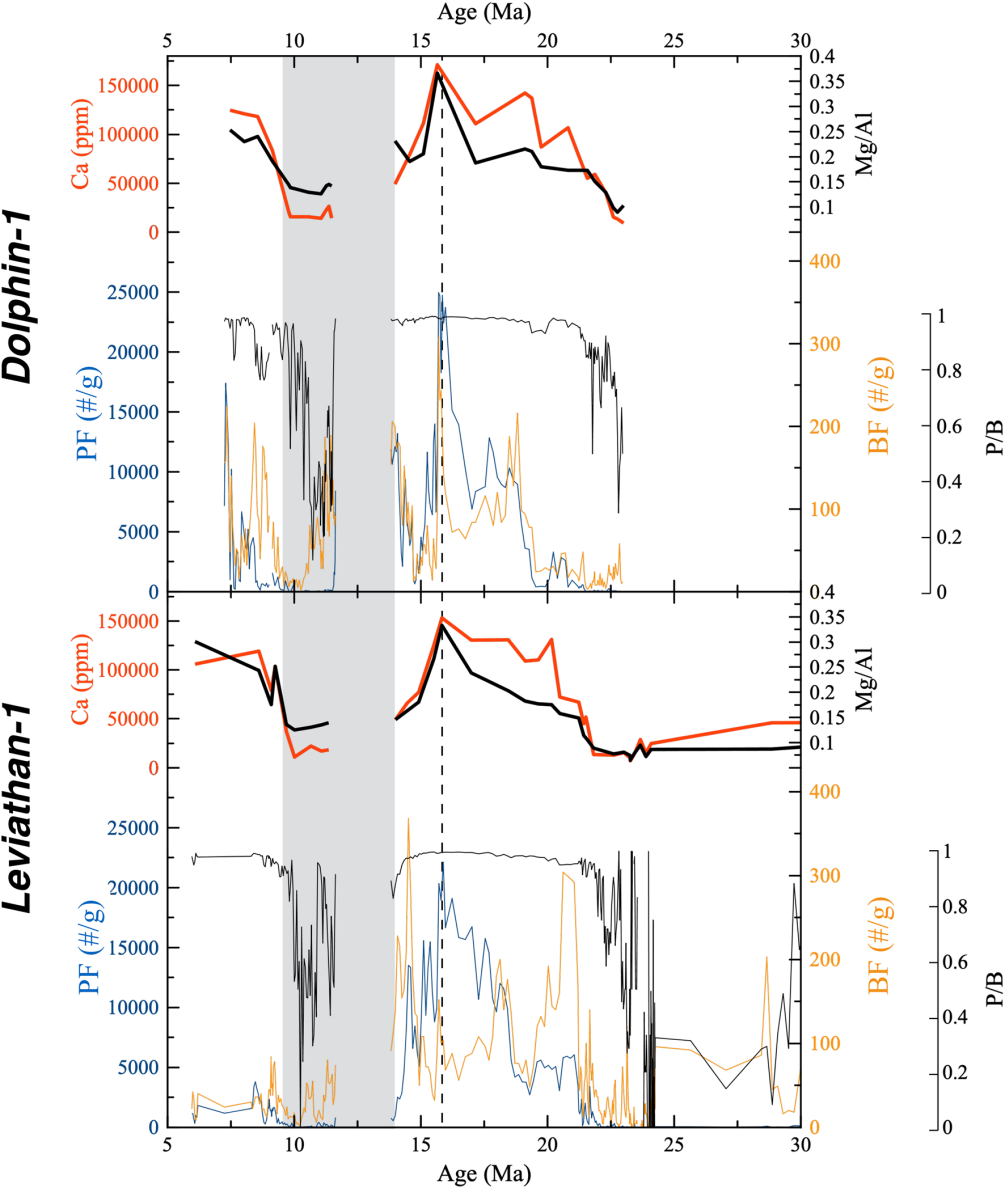
Foraminifera abundances, Ca concentrations and Mg/Al ratios. A drop in the planktonic to bentic ratio (P/B) reflects bottom carbonate dissolution. Ca concentrations trace the CaCO_3_ content (see Fig. S2), which are in phase with Mg/Al ratios and foraminifera abundances. A vertical dashed curve marks the peak of the MMCO (~ 16 Ma). A grey rectangle marks the timing of the carbonate crash in the PM.

**Figure 5:**
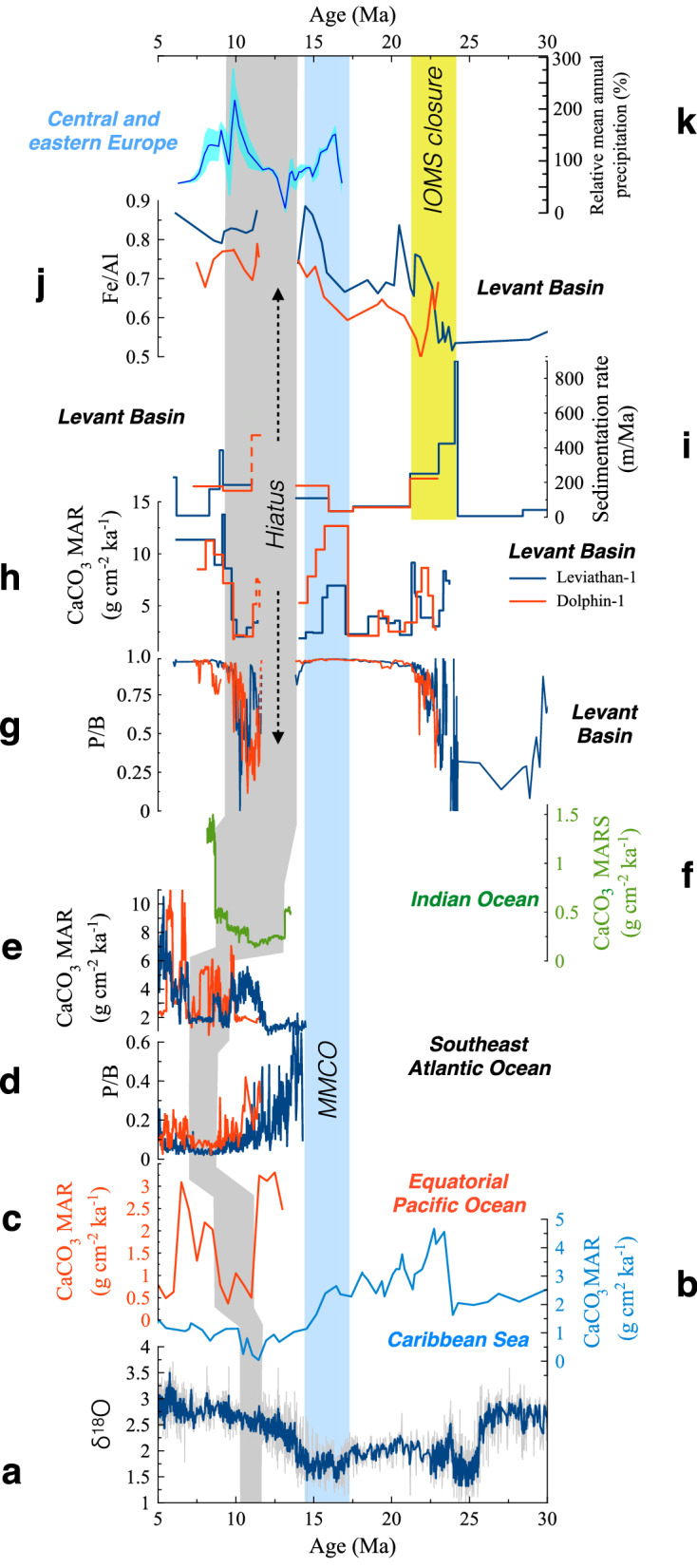
Global and regional climate records. (**a**) Globally stacked benthic δ^18^O^50^, CaCO_3_ MARs in the (**b**) Caribbean Sea^51^, (**c**) East equatorial Pacific Ocean, site 572^36^, (**d**) P/B ratio at southeast Atlantic Ocean, sites 1085 (red) and 1087 (blue)^38^, (**e**) CaCO_3_ MARs at southeast Atlantic Ocean, sites 1085 (red) and 1087 (blue)^38^, (**f**) CaCO_3_ MARs at equatorial Indian Ocean^39^. Levant Basin records (Leviathan-1 in blue and Dolphin-1 in red): (**g**) P/B ratios, (**h**) CaCO_3_ MARs, (**i**) Linear Sedimentation Rates (m/Ma), (**j**) Fe/Al ratios, and (**k**) relative mean annual precipitation (%) in central and eastern Europe (light blue shading marks the 1σ confidence interval)^47^. A yellow rectangle marks the timing of the primary closure of the IOMS, as determined here, a light blue rectangle marks the timing of the MMCO and coeval pulse in productivity and increased CaCO_3_ MARs in the proto Mediterranean Sea, and a grey rectangle marks the timing of MCC events in different regions. Note that additional MAR records are available in the Pacific Ocean^36^ but were not shown in order to maintain clarity.

Both sites show a remarkably good fit between chronostratigraphic units and the major element chemostratigraphy (Figs. 2, 3, 4), whereby the bulk chemical composition is dominated by cyclic fluctuations in CaCO_3_ ranging between ~ 5 and 35% (Fig. S2). Planktonic and bentic foraminifera counts show strong variations with a collapse in the shell abundances and the planktonic/bentic ratio (P/B, reflecting the fraction of planktic forams in the total of planktic plus benthic forams) during the Aquitanian and Chattian, as well as during the early Tortonian, compared to an intermediate peak during the Langhian, corresponding with the MMCO (~ 17–15 Ma) (Fig. 4). The bulk Fe/Al ratios range between ~ 0.55 during the Chattian, slightly higher than upper continental crust (UCC) values (0.43^17^), to ~ 0.7–0.85 during the late Miocene (Fig. 5j).

### Arabian–Eurasian collision

The abrupt pulse of massive terrigenous sediment influx and deposition during 24–21 Ma (Figs. 3, 5i) has important implications on our understanding of the Cenozoic evolution of the triple plate junction of the Arabia, Africa, and Eurasian plates, the depositional regime that controlled the Levant Basin, and regional paleogeography. As mentioned, the timing of closure of the IOMS is currently very poorly constrained. Previous estimates pertain to the mid Oligocene to early Miocene (~ 30–20 Ma) based on reconstructions of the Arabia–Eurasia tectonic collision^10,11,18^, though other paleogeographic and biostratigraphic reconstructions have associated the closure of the IOMS with significantly younger stages between the mid to late Miocene^19–24^. Here, we fine-tune the wide age range and interpret the pulse of MARs between 24 and 21 Ma to indicate an increase in continental weathering and influx of terrigenous erosional products, concomitant, and related to, regional doming (Cairo plume) and uplift associated with the Red Sea rifting, in itself corresponding with a northward shift of the Arabian plate^25,26^ towards, and eventually colliding with the Eurasian plate. Thus, the Indian Ocean became effectively isolated from the PM, even if a shallow pathway still existed after 21 Ma. This conclusion is in accord with the results of a recent study that suggested, based on εNd compositions of sections in the Indian Ocean and Mediterranean Sea, that water mass exchange across the IOMS was reduced by ~ 90% ca. 20 Ma^27^, only briefly later than estimated here.

Following the rifting and uplift, which provided for the abundant supply of erosional products to be transported to the basin via the Nile Delta, we postulate that the PM was strongly effected by a compressional event at ~ 17 Ma. This event created the NE-SW anticlines that have been the focus of natural gas exploration over the past 15 years (i.e. Leviathan, Tamar, Aphrodite, Tanin, Karish, Dalit fields), as well as (re)activated the Temsah-Bardawil trend, which in turn blocked the influx of sand from the Nile Delta into the Levant Basin (Fig. S3). As the structural setting between the Leviathan and Dolphin sites differ (i.e. Leviathan being a high-standing compressional anticline and Dolphin being a faulted block in an area that was not uplifted, Fig. 2), their evolution slightly differs after 17 Ma, though the primary oceanographic signatures remain similar (Figs. 3, 5).

### Miocene climate optimum and carbonate crash

Following the effective closure of the IOMS by ~ 20–21 Ma, the Levant Basin experienced an increase in oceanic productivity, that peaked during the Langhian (~ 17–15 Ma) as reflected by high planktonic and bentic foraminifera abundances and P/B ratio (Figs. 4, 5), coinciding with the globally observed Mid-Miocene Climate Optimum (MMCO), when a warm perturbation induced higher primary productivity rates globally^1,28^.

Following the MMCO, a ~ 2 Myr hiatus corresponding with the Serravallian, is capped by a ~ 500-m-thick Tortonian interval (Fig. 2) that consists mainly of shales, with minimum contents of foraminifera and CaCO_3_, as well as a drop in P/B ratios (Fig. 5, Table S3), indicative of carbonate dissolution rather than a decrease in productivity^29,30^. Indeed, the sedimentary hiatus across the Serravallian is well documented in previous studies of marginal sites from this region^7,31–34^, and although it was previously assumed to represent a sea level drawdown associated with the glaciation of Antarctica^27^, its occurrence in the deepwater Leviathan-1 and Dolphin-1 wells suggests the impact of additional processes. The approximate Mid-Miocene water depth at this site is estimated to be ~ 2.5 km^35^ and hence it is unreasonable that this hiatus reflects subaerial exposure nor is it likely that the deepest part of the basin experienced significant sediment winnowing, particularly preferential winnowing of CaCO_3_. By contrast, the ~ 13–10 Ma time interval corresponding with the hiatus, low CaCO_3_ contents and drop in P/B ratios (Fig. 5), coincides and slightly precedes a globally scaled Miocene Carbonate Crash (MCC) which was first identified in the Pacific Ocean and the Caribbean Sea^21,30,36,37^, and has since been reported in the Atlantic Ocean^29,38^ and Indian Ocean^39^.

Although the triggers and timing of the MCC are still largely unresolved, it has been suggested^29,40^ that it was driven by a combined change in primary production rates and concomitant bottom carbonate dissolution associated with a reorganization of global thermohaline circulation. The latter has been suggested to be induced in the Pacific Ocean and Caribbean Sea by a reduction in deep-water exchange between the Atlantic and Pacific Oceans across the Panama Gateway, that resulted in the re-establishment and intensification of the North Atlantic Deep Water production and coeval influx of corrosive southern-sourced intermediate waters into the equatorial Pacific Ocean^30,36^. By contrast, this event had also been suggested to be driven by changes in the intensity of chemical weathering and riverine input of calcium and carbonate ions into the oceans^39^. These suggestions are largely based on an assumption of an overlap in timing between the various global expressions of the MCC, which therefore have a common cause^30^. As new observations accumulate, this assumption appears less likely. Indeed, the onset and termination of the MCC at the Pacific, Caribbean, Atlantic and Indian Oceans range between ~ 11–8, 12–10, 9.6–7 and 13.1–8.7 Ma, respectively^29,30,36,38,39^, compared with 13.8–9.5 Ma at the PM. Assuming these temporal offsets do not stem from chronological biases, it becomes likely that a core interval ca. 11–10 Ma was indeed a time of a global carbonate crash in the oceans, but that this event was strongly influenced by regional processes that determined its onset and longevity, which differed between regions. We note that at this stage (Tortonian), the PM is already relatively isolated and while the connection to the Atlantic Ocean exists, it is very restricted (Fig. 1).

### Regional drivers or global connections?

The exact trigger of the dissolution process in the late Miocene PM is not clear, nor is it entirely plausible that all of the globally identified carbonate crash events are modulated, even if only partially, by the same single mechanism. Water depths at the study site have been estimated to be approximately 2.5 km during the Miocene^35^, well above the carbonate compensation depth (CCD). Indeed, it is important to emphasize that while the reduction in CaCO_3_ MARs in deep open ocean environments can be driven by the cumulative effects of dissolution and decrease in CaCO_3_ production, the observed drop in P/B ratios, together with the sedimentary hiatus across the Serravallian interval, suggest that the crash in CaCO_3_ MARs must be strongly controlled by dissolution processes.

To account for this, we consider the role of the Paratethys (Fig. 1), located north of the PM, whose shallow but widespread inland water bodies were likely Fe-enriched due to their contact with marsh-like environments, with typical anoxic conditions that favored the release of dissolved Fe from the sediment to the interstitial and overlaying waters. The spill out of Paratethys waters into the PM, which was probably more intense during regionally wet periods, involved the influx of excess Fe and other nutrients. The former was scavenged as Fe-oxides across the PM water column and removed to the seafloor, as reflected by the increased Fe/Al ratios (Fig. 5j), which exceed average upper continental crust values (Fig. S1). Accordingly, the inflow of Paratethys waters probably supported increased primary productivity and enhanced export production of organic material to the seafloor. The oxic decomposition of organic matter lowers pH and can drive CaCO_3_ dissolution in sediments^41^ and even along the water column, as shallow as ~ 1 km^42^. Indeed, this mechanism of CaCO_3_ dissolution is well documented across several sapropel layers in the Mediterranean Sea since the Miocene^43,44^. By inference, this suggests that the decomposition of organic matter triggered massive bottom carbonate dissolution in the Levant Basin during 13.8–9.5 Ma. This also implies that bottom oxygen could not have been fully consumed by the decomposition process because otherwise the development of anoxic conditions would have favored carbonate preservation^45^. Moreover, high P/B ratios observed between the IOMS closure and the end of the MMCO (~ 21–13.8 Ma; Fig. 5g) indicate negligible carbonate dissolution, while an increase in planktonic foraminifera reflects a gradual increase in productivity (Fig. 4). A later interval of high P/B ratios that occurred after 9.5 Ma is interpreted to mark the end of the carbonate dissolution interval though planktonic foraminifera counts remain at intermediate levels, reflecting weak productivity relative to ~ 21–13.8 Ma.

These patterns correspond with coeval climate reconstructions across the Paratethys (Fig. 5k). Regionally, continental records of the ecophysiological structure of herpetological assemblages (amphibians and reptiles) from southwest, central and eastern Europe^46,47^ have indicated the dominance of dry conditions across Europe between 13 and 11 Ma, corresponding with the Serravallian hiatus in the PM, followed by a so-called “washhouse” wet climate that persisted between 10.2 and 8.5 Ma. During the former period, dry conditions would have limited the riverine influx and hence reduced the combined contribution of alkalinity to the PM, resulting in reduced CaCO_3_ MARs. More importantly, the reduced influx of Paratethys waters would weaken water column stratification and result in enhanced oxic decomposition of organic matter in the sediment–water interface, with a corresponding increase in bottom CaCO_3_ dissolution. This effect would have been reversed in response to the subsequent “washhouse” interval, which indeed, corresponds to a rise in CaCO_3_ MARs (Fig. 5h). The PM is therefore characterized by intense CaCO_3_ dissolution during the Serravalian and lower Tortonian that together with widespread subaerial exposure of marginal sequences due to sea level drop associated with the glaciation of Antarctica^27^, resulted in destabilization of the sedimentary column, as indicated by the lack of coherent reflections and internal seismic architecture across the Tortonian section (Fig. 2).

It is further worth considering the role of Africa in modulating oceanographic conditions in the PM. Climate models have implied that the IOMS closure, which we determined to have taken place during the Aquitanian, triggered a reorganization of atmospheric moisture transport leading to a significant reduction in North African precipitation and a subsequent aridification and development of the Sahara Desert^22^. The combined effect of the latter would be a gradual long term reduction in the supply of alkalinity from North Africa to the PM, which would support the dissolution of marine carbonates.

Given its relative isolation, the PM history displays a surprisingly strong correlation with global climate patterns. While it is readily acknowledged that these connections are not fully understood, they do reflect the role of the PM record as a climatic amplifier and hence open up the possibility for a detailed investigation and reconstruction of subtropical continental climate trends during the Miocene, particularly after the IOMS closure ca. 21 Ma. Moreover, the cumulative evidence for the wide spread global onset of carbonate crash episodes during the late Miocene, despite their temporal offsets and *a-priori* regionally driven mechanisms, implies that their occurrence is not coincidental and is modulated by a wide scale process that we postulate is related to global changes in chemical weathering and alkalinity supply to the oceans.

## Methods

### Well data

The Leviathan-1 (LIV) and Dolphin-1 (DOL) exploration wells were drilled in water depths of ~ 1600 m, and are located approximately 120 km west of Haifa, Israel (Dolphin well (N 3628144.05 m, E 575444.97 m; coordinates in UTM 36 N WGS 1984), Leviathan-1 (N 3653455.35 m, E 553663.40 m); Fig. 1). Both wells were designed and drilled to explore the potential of Late Tertiary natural gas reservoirs. The lithology of both wells is quite similar, with DOL displaying slightly thicker sections as it was drilled down-dip. The original well forecast was built based on high quality seismic sections and the findings of the drilling of the Tamar gas field, previously drilled by Noble Energy and partners. Both wells were drilled with salt saturated (composed principally of potassium chloride and barite) drilling mud.

### Samples and processing

Cutting samples were collected every 9 m until a depth of 4527 m and at an interval of every 3 m from that depth until the end of the well at a depth of 6261 m in the Leviathan-1 well. In the Dolphin-1 well, samples were collected at an interval of 9 m until a depth of 3351 m and at an interval of every 3 m until a final depth of 5725 m. These samples were washed and defined on the drilling rig. Biostratigraphic analysis, which was the basis for the updated age model presented here, was performed in house by the operator of these wells, Noble Energy Inc. Sixty-two of these well cuttings samples (and one sample of drill mud used during the coring operations) were gently homogenized by grounding with a mortar and pestle, and filtered through a 63 µm sieve. About 100 mg of the smaller size fraction samples were weighted into a Teflon beaker and digested in a mixture of double-distilled concentrated HNO_3_-HF-HClO_4_ through several digestion cycles until full dissolution was achieved. The solutions were then dried down, brought up in 4 N HNO_3_, and a secondary 1:2000 dilution was prepared using 3% HNO_3_ as a matrix. The abundances of major elements were then determined on an Agilent 7500cx Inductively Coupled Plasma Mass Spectrometer (ICPMS) and the Institute of Earth Sciences, Hebrew University of Jerusalem. Gravimetrically prepared standard solutions were used to calculate linear calibration curves for the measured element intensities. In addition, the measurements of the elemental abundances of several splits of two geologic standard materials (BCR2 and BHVO-1) were used to improve the accuracy of the results. Procedural blanks were routinely monitored.

The sensitivity of the results to possible biases inserted by the presence of drill mud was evaluated through a mass balance simulation where the measured elemental abundances were assumed to represent a mixture of 95% original sample and 5% drill mud. Correcting for the presence of drill mud (using its measured values) the elemental concentrations were recalculated for all samples. The differences between the measured and corrected values are negligible, with no effect on the following discussion and conclusions.

Figure S1 compares between the sample compositions and that of the drill mud. Evidently, the drill mud is distinct from that of the samples, and no tailing is identified in the latter, which could have indicated a significant contamination of the samples. Moreover, the secular variations in Ca and Mg/Al (Fig. S1c,d) are independent of the drill mud composition and *a-priori* range between two natural end members.

CaCO_3_ concentrations were measured using a calcimeter. The results correspond with the measured Ca concentrations (Fig. S2) allowing for their use as carbonate content proxies. The latter are reported in Table S2.

### Mass accumulation rates

Mass accumulation rates of bulk samples and carbonates (Fig. 3) were calculated by multiplying linear sedimentation rates (LSRs) derived from age–depth models, with the dry bulk density derived from wireline logging (which calculates bulk density based on Gamma Ray scatter at varying depths in the wellbore) and the carbonate weight percentage (% CaCO_3_) as determined here by direct analyses using the calcimeter or by converting from measured Ca concentrations (e.g. Fig. S2).

## Supplementary information

Supplementary Information 1.

Supplementary Information 2.
