# Supplementary material for: The Oligo–Miocene closure of the Tethys Ocean and evolution of the proto-Mediterranean Sea
Source: Sci Rep. 2020 Aug 14;10:13817. doi: 10.1038/s41598-020-70652-4 (PMC7427807; doi:10.1038/s41598-020-70652-4)
Supplement: Supplementary file 1 — Supplementary Information 1. [file 41598_2020_70652_MOESM1_ESM.pdf]

Supplementary material

The Oligo-Miocene closure of the Tethys Ocean and evolution of the proto-Mediterranean Sea

Adi Torfstein[1,2,]* and Josh Steinberg[3]

[1] The Fredy & Nadine Herrmann Institute of Earth Sciences, The Hebrew University of Jerusalem, Jerusalem 91904, Israel.

[2] The Interuniversity Institute of Marine Sciences of Eilat, Israel.

[3] Ratio Oil Exploration, Tel Aviv, Israel

* correspondence: Adi.torf@mail.huji.ac.il

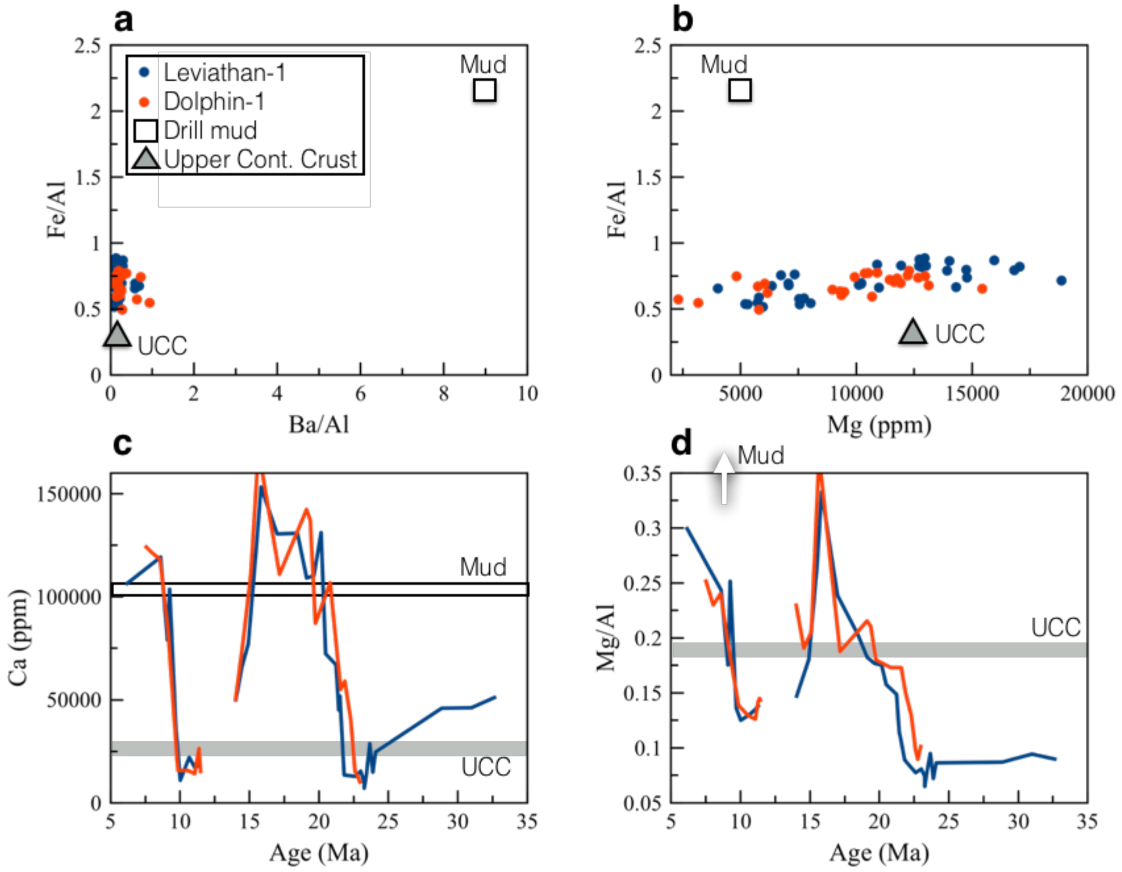

Figure S1| Elemental abundances in Leviathan-1 (red) and Dolphin-1 (blue) wells. The sample compositions display variations independent of the upper crust composition (UCC) and of the composition of drill mud used in their extraction, implying that the methodological treatment of the samples, together with appropriate choice of proxies that are not likely to be contaminated by the drill mud (e.g., Fe, Al, Ca), allows assuming the results are robust tracers of natural processes in the PM.

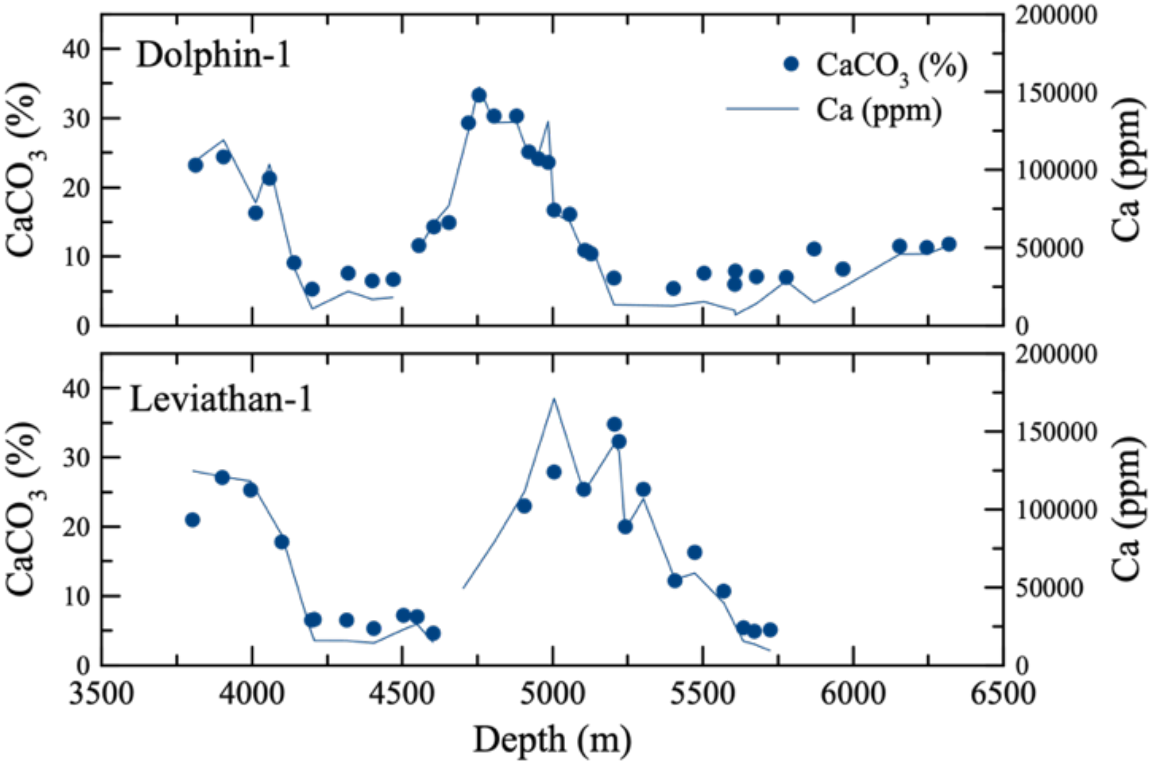

Figure S2| Comparison between $CaCO_3$ and Calcium concentrations.

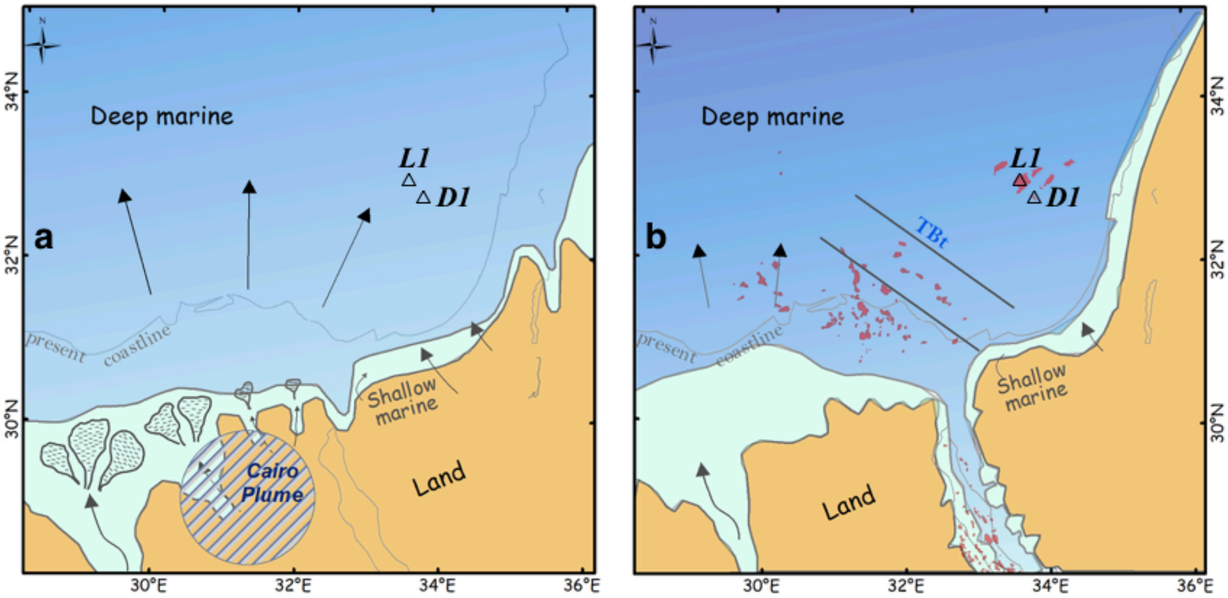

Figure S3| Reconstruction of the tectonic setting in the proto- eastern Mediterranean. (a) 24-21 Ma; uplift of Cairo and Afar plumes that drove massive sedimentation in the Levant Basin. Reconstructed paleogeography taken from Salem[6]; Gvirtzman et al.[7]; Steinberg et al.[8]; Bosworth et al.[1] (ArcMap software v10.5, https://www.esri.com/en-us/arcgis/products/arcgis-pro/overview), (b) 17 Ma; evolution of compressional features and activation of the Temsah Bardawil trend (TBt), which limited sand influx into the Levant Basin. Red polygons mark the Oligo Miocene natural gas fields that represent compressional structures. Reconstructed paleogeography taken from: Salem[6]; Gvirtzman et al.[7]; Steinberg et al.[8].

Table captions

Table S1| Anchor points used for development of the age models for Leviathan-1 and Dolphin-1.

Table S2| Elemental abundances and $CaCO_3$ concentrations for Leviathan-1 and Dolphin-1.

Table S3| Planktonic and bentic counts at Leviathan-1 and Dolphin-1.

References

1. Bosworth, W. *The Red Sea*. (2015). doi:10.1007/978-3-662-45201-1

2. Segev, A., Avni, Y., Shahar, J. & Wald, R. Late Oligocene and Miocene different seaways to the Red Sea–Gulf of Suez rift and the Gulf of Aqaba–Dead Sea basins. *Earth-Science Rev.* **171**, 196–219 (2017).

3. Avni, Y., Segev, A. & Ginat, H. Oligocene regional denudation of the northern Afar dome: Pre- and syn-breakup stages of the Afro-Arabian plate. *Bull. Geol. Soc. Am.* **124**, 1871–1897 (2012).

4. Rogl, F. Mediterranean and Paratethys. Facts and hypotheses of an Oligocene to Miocene paleogeography (short overview). *Geol. Carpathica* **50**, 339–349 (1999).

5. Bialik, O. M., Frank, M., Betzler, C., Zammit, R. & Waldmann, N. D. Two-step closure of the Miocene Indian Ocean Gateway to the Mediterranean. *Sci. Rep.* **9**, 8842 (2019).

6. Rafik Salem. Evolution of Eocene-Miocene Sedimentation Patterns in Parts of Northern Egypt. *Am. Assoc. Pet. Geol. Bull.* **60**, (1976).

7. Gvirtzman, Z. *et al.* Retreating Late Tertiary shorelines in Israel: Implications for the exposure of north Arabia and Levant during Neotethys closure. *Lithosphere* **3**, 95–109 (2011).

8. Steinberg, J., Gvirtzman, Z., Folkman, Y. & Garfunkel, Z. Origin and nature of the rapid late Tertiary filling of the Levant Basin. *Geology* **39**, 355–358 (2011).
